# Case report: Rapid resolution of grade IV ICANS after first line intrathecal chemotherapy with methotrexate, cytarabine and dexamethasone

**DOI:** 10.3389/fimmu.2024.1380451

**Published:** 2024-05-03

**Authors:** Mikalai Katsin, Tatsiana Shman, Alexandr Migas, Dzmitry Lutskovich, Yuliya Serada, Yauheniya Khalankova, Yuliya Kostina, Simon Dubovik

**Affiliations:** ^1^ Department of Hematology, Vitebsk Regional Clinical Cancer Centre, Vitebsk, Belarus; ^2^ Laboratory of Genetic Biotechnologies, Belarusian Research Center for Pediatric Oncology, Hematology and Immunology, Minsk, Belarus; ^3^ Laboratory of Molecular Diagnostics and Biotechnology, Institute of Bioorganic Chemistry of the National Academy of Sciences of Belarus, Minsk, Belarus

**Keywords:** ICANS, intrathecal chemotherapy, CD19 CAR-T cells, anakinra, corticosteroids

## Abstract

Corticosteroid therapy is the mainstay of immune effector cell-associated neurotoxicity syndrome (ICANS) management, although its use has been associated with worse overall survival (OS) and progression-free survival (PFS) after chimeric antigen receptor T-cell (CAR-T cell) therapy. Many options are being investigated for prophylaxis and management. Accumulating evidence supports the use of intrathecal (IT) chemotherapy for the management of high-grade ICANS. Here, we describe a case of a patient with stage IV Primary mediastinal B-cell lymphoma (PMBCL) successfully treated with IT methotrexate, cytarabine, and dexamethasone as first-line therapy for CD19 CAR-T cell-associated grade IV ICANS. The stable and rapid resolution of ICANS to grade 0 allowed us to discontinue systemic corticosteroid use, avoiding CAR-T cells ablation and ensuring preservation of CAR-T cell function. The described patient achieved a complete radiologic and clinical response to CD19 CAR-T cell therapy and remains disease-free after 9 months. This case demonstrates a promising example of how IT chemotherapy could be used as first-line treatment for the management of high-grade ICANS.

## Introduction

Chimeric antigen receptor T-cell (CAR-T cell) therapy has recently emerged as a novel treatment modality for the management of B-cell acute lymphoblastic leukemia, non-Hodgkin lymphoma, and multiple myeloma, with high response rates and a potential for cure. To date, the Food and Drug Administration has approved six CAR-T cell products for different indications, and numerous CAR-T cell trials are being widely carried out. Despite great clinical success, complications such as cytokine release syndrome (CRS) and immune effector cell-associated neurotoxicity syndrome (ICANS) can be fatal and can pose obstacles in the clinical application of CAR-T cells. Anti-IL-6 receptor antibody (Tocilizumab) and corticosteroid therapy are the mainstream management strategies for CRS and ICANS, respectively (1). It was recently reported that a high cumulative corticosteroid dose, especially in high-grade ICANS, is associated with worse overall survival (OS) and progression-free survival (PFS) after CAR-T cell therapy due to the inhibition of CAR-T cell function and persistence (2). Various prophylaxis and preemptive strategies, such as early (3) and prophylactic (4) glucocorticosteroid administration, IL-1 receptor antagonist (Anakinra), anti-GM-CSF antibodies (Lenzilumab) and ibrutinib incorporation into the backbone of the CAR-T cell protocol (5), are being explored and have shown some evidence of success. Nevertheless, in cases of ICANS occurrence, strategies aimed at reducing the cumulative corticosteroid dose could impede systemic CAR-T cell depletion and on improve OS and PFS by controlling the pathological inflammatory response in the central nervous system (CNS). We report a case of relapsed/refractory (R/R) stage IV Primary Mediastinal B-Cell Lymphoma (PMBCL) developing late grade IV ICANS after CD19 CAR-T cell therapy. Intrathecal (IT) administration of methotrexate, cytarabine, and dexamethasone as first-line therapy successfully resolved ICANS IV within one day. The rapid response allowed us to discontinue intravenous (IV) dexamethasone and ensure CAR-T cell functional activity. The patient reached a sustained complete response according to positron emission tomography–computed tomography (PET-CT), which has lasted for over 9 months.

## Clinical case description

**Figure 1:**
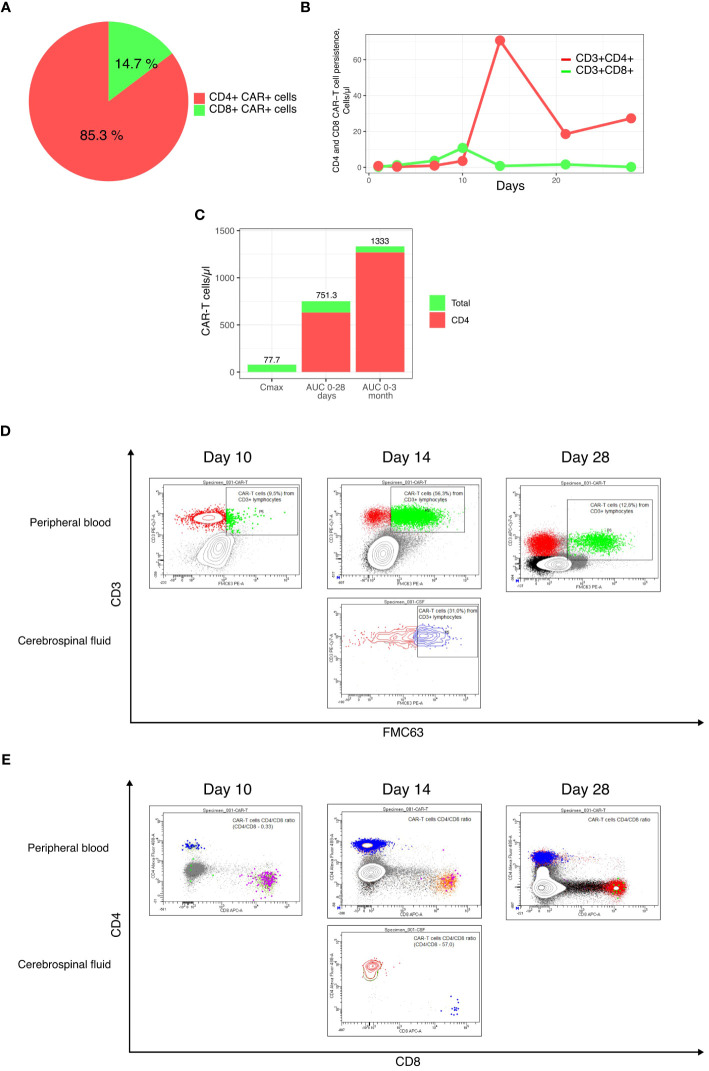
Final CAR-T cell product and CAR-T cell subsets persistence characteristics. **(A)** CD4+ and CD8+ subsets composition of the final CAR-T cell product; **(B)** Persistence of CD4+ and CD8+ CAR-T cells; **(C)** Cmax, AUC0-28 days and AUC0-3 months; **(D, E)** Flow cytometry plots of CAR-T cells in the peripheral blood and cerebrospinal fluid.

**Figure 2:**
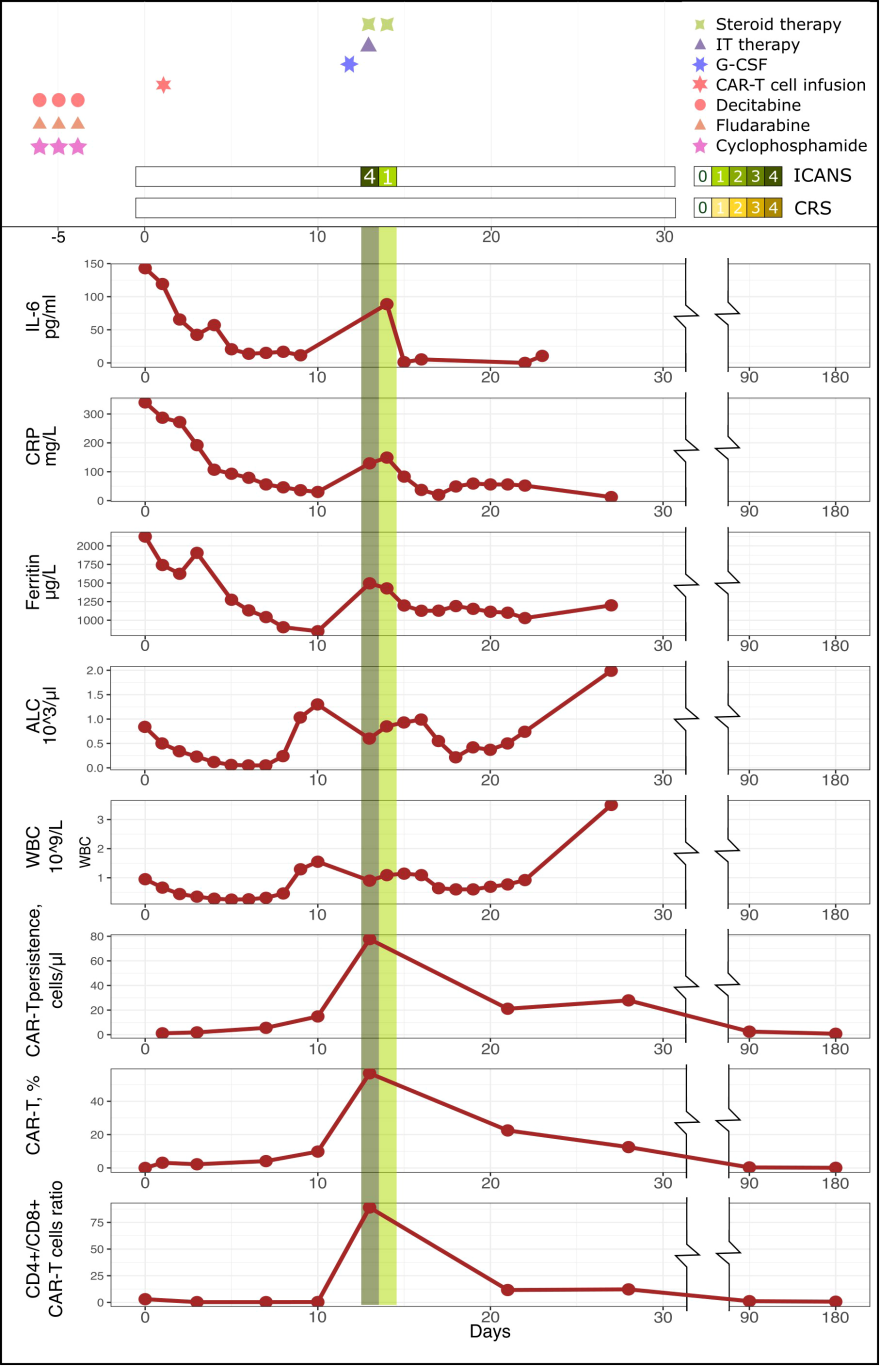
Biochemical and immunological kinetics after CD19 CAR-T cell infusion.

A 39-year-old male patient was admitted to our department with R/R stage IV PMBCL involving the mediastinum (170 mm), pleura, lungs, bone marrow, and lymph nodes from both sides of the diaphragm, along with dyspnea and B-symptoms. He was refractory to R-DA-EPOCH, R-MACOP-B, and Nivo+Bv. His blood CD3+ cell count was 1220.43 cells/μL, and leukapheresis was successfully performed. A lentiviral vector containing a second-generation CAR was constructed based on an expression cassette coding for a CD19-specific single-chain variable fragment (FMC63) fused to the IgG4 hinge, CD28 transmembrane domain, 4-1BB, and CD3z cytoplasmic signaling domains. Additionally, a truncated version of EGFR (EGFRt) was added after the P2A sequence as a surface marker for CAR-T cell tracking. IL-7 and IL-15 were used for CAR-T cells expansion. Following ex vivo lentiviral transduction and expansion, CAR-T cells totaling approximately 1.29 * 10^6^ cells were obtained, with CD4+ CAR-T cells constituting 85.3% (**Figure 1A**). Restaging with PET-CT and subsequent lymphodepletion (decitabine, cyclophosphamide, and fludarabine) were performed. High-risk factors included a high intermediate age-adjusted International Prognostic Index -2, bulky disease, CRP 340 mg/L (6), and a CAR-HEMATOTOX score of 2 (7). After 7 days of CAR-T cell infusion, the patient’s symptoms improved, dyspnea resolved, inflammatory markers returned to normal values, and no signs of CRS or ICANS were registered in the first 10 days (see **Figure 2**). However, on day +14, the patient developed grade III ICANS with an Immune Effector Cell-Associated Encephalopathy (ICE) score of 2 points. No concomitant CRS has been observed. After the diagnosis of grade III ICANS, IV dexamethasone 20 mg was immediately initiated. A brain computed tomography (CT) scan did not reveal any intracranial abnormality, and an opportunity to perform an electroencephalography study was not available. Serum concentrations of inflammatory markers also significantly increased, and CAR-T cells reached Cmax at 56.3% (77.7 cells/μL) on day +14 (**Figures 1C**, **2**).

**Figure 3:**
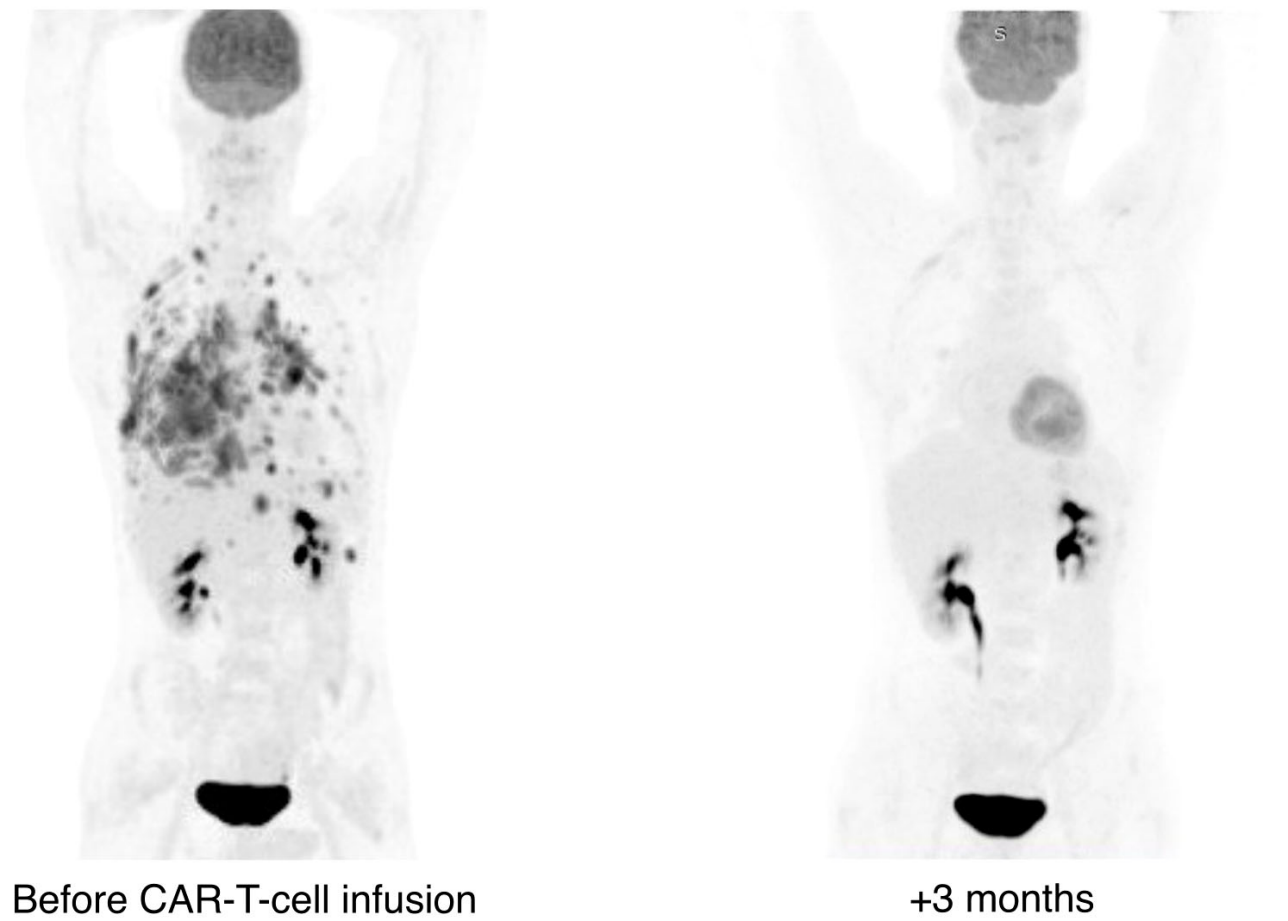
Radiological scans during CD19 CAR-T cell therapy.

In our case, CAR-T cell expansion was primarily achieved through CD4+ CAR-T cells, resulting in a CD4+/CD8+ ratio of 89 (**Figures 1B**, **2**). Within 1 hour after the initial dose of IV dexamethasone, the patient rapidly deteriorated, progressing to a comatose level of consciousness and non-responsiveness to tactile or auditory stimuli, consistent with ICANS grade IV. The patient did not require intubation or mechanical ventilation. A lumbar puncture was performed, revealing a normal opening pressure. Cerebrospinal fluid analysis ruled out viral and bacterial infections and indicated a white blood cell count of 2 cells/μl. CAR-T cells comprised 31.6% of leukocytes in the cerebrospinal fluid, with a CD4+/CD8+ CAR-T cell ratio of 54 (**Figures 1D, E**). In view of the low dose of CAR-T cells infused and the patient’s high-risk disease factors, first-line IT chemotherapy (methotrexate 15 mg, Ara-C 40 mg, dexamethasone 4 mg) was immediately implemented after the onset of grade IV ICANS to preserve CAR-T cell function. Within 6 hours of the IT chemotherapy, the patient showed a dramatic clinical improvement, with ICANS decreasing to grade II (ICE 6 points). In less than 32 hours after the IT chemotherapy, the patient’s ICANS had resolved to grade 0, inflammatory markers decreased, and steroid therapy was discontinued to ensure CAR-T cell function (**Figure 2**). The cumulative steroid dose was 96 mg of IV dexamethasone. CAR-T cells continued to be detected at significant levels in the peripheral blood (+28 days - 27.9 cells/μl, +3 months – 2.5 cells/μl, +6 months – 0.76 cells/μl), maintaining B-cells aplasia (**Figures 1D, E**, **2**). PET/CT scans at +3 months (**Figure 3**) and CT scans at +6 and +9 months confirmed an ongoing complete remission (CR). The area under the curve (AUC) for 0-28 days of CAR-T cells amounted to 751.3 cells/μl, including CD4+CAR-T cells 632.2 cells/μl. The AUC for 0-3 months amounted to 1333 cells/μl, including CD4+ CAR-T cells 1269.4 cells/μl (**Figure 1C**).

## Discussion

ICANS is a potentially serious complication of CAR-T cell therapy, encompassing symptoms ranging from mild encephalopathy and disorientation to more severe and potentially fatal manifestations such as acute cerebral edema, paresis, plegia, seizures, and loss of consciousness. Approximately half of patients with diffuse large B-cell lymphoma experience ICANS, with 70% encountering any grade and 35% experiencing grade ≥3 ICANS following CD28-based anti-CD19 CAR-T cells (8). Conversely, 4-1bb-based anti-CD19 CAR-T cells are associated with a lower incidence and severity of ICANS, with 26% experiencing any grade and 10% experiencing grade ≥3 ICANS (9). In most cases, acute ICANS develops 4–6 days after CAR-T cell infusion and typically occurs after the onset of CRS or in the setting of improving or resolved CRS (10). Late-onset ICANS cases have also been reported (11, 12), lasting 5–13 days with complete symptom resolution in the majority of cases (13, 14). Clinical studies have identified IL-6, GM-CSF, IFN-γ, and IL-15 as major cytokines responsible for CNS inflammation after CAR-T cell therapy (15). While IL-1 hasn’t displayed a strong association with ICANS in clinical studies, anti-IL-1R antibodies have been shown to prevent ICANS in preclinical mouse models of CAR-T cell therapy (16). CAR-T cells produce IFN-γ, TNF-α, and GM-CSF at the tumor site to attract and activate macrophages/monocytes, which in turn produce large amounts of IL-1, IL-6, nitric oxide, and other cytokines associated with CRS presentation. The abundance of cytokines and soluble inflammatory mediators in the circulation leads to the activation of CNS endothelium and disruption of the blood-brain barrier (BBB), driven by CAR-T cells themselves and amplified by activated macrophages (17). Consequently, the severity of CRS is considered a predictor of neurotoxicity development in the clinical context (18). Subsequently, the diffusion of cytokines, migration of CAR-T cells and peripherally activated monocytes into the CNS, resident microglia activation, and neuronal cell injury occur. However, not all ICANS cases are preceded by CRS. Another mechanism of neuroinflammation and BBB disruption is the direct production of cytokines and mediators within the CNS (19). One additional pathophysiological mechanism of ICANS, particularly cerebral edema, could result from significant disruption of the BBB with a massive influx of cytokines, inflammatory mediators, and plasma leakage into the brain, even without any detection of CAR-T cells in the CNS (20).

The composition of CAR-T cell subpopulations in the apheresis material and the final CAR-T cell product may indeed impact the efficacy and toxicity of CAR-T cells. In the majority of cases, patients with B-cell malignancies tend to have a higher proportion of CD8+ T cells in the peripheral blood at the time of apheresis (21). CD4+ CAR-T cells have been shown to produce increased amounts of cytokines such as IFN-γ, TNF-α, and IL-2. Additionally, they demonstrate a robust proliferative capacity upon the recognition of tumor cells and tend to have a lesser degree of exhaustion, resulting in longer persistence (22–24). Baur et al. (25) investigated the contribution of CD4+ and CD8+ CAR-T cell expansion kinetics to the clinical response and the development of toxicities. They found a positive correlation between the AUC for CD4+ CAR-T cells at 0-28 days and 0-3 months and the development of CR at 1 and 3 months, respectively. The severity of CRS and ICANS also positively correlated with the AUC for CD4+ CAR-T cells at 0-28 days (25). This case is in concordance with the findings of the study, as the patient developed grade IV ICANS at the peak of CD4+ CAR-T cell expansion and eventually achieved a CR, largely due to the CD4+ CAR-T cell subset.

The majority of cases of ICANS are a consequence of hyperinflammation outside the CNS, making prophylaxis a key strategy to reduce its incidence and severity. Current prophylaxis options include steroids, anakinra, and JAK inhibitors (26). Clinical studies of the anti-GM-CSF antibody Lenzilumab are also underway, with results pending (27, 28). Anakinra can cross the BBB when given intravenously, although its large molecular size allows only 4% penetration into cerebrospinal fluid with standard dosing regimens (29). This suggests that Anakinra could be much more effective if prescribed earlier in the clinical scenario with the aim of preventing endothelial disruption and ICANS development. In a Phase II study of Anakinra prophylaxis, all-grade ICANS occurred in 19%, and severe ICANS occurred in 9.7% of patients (30). Another option for ICANS prophylaxis is the utilization of steroids. In the ZUMA-1 cohort study (NCT02348216), 40 patients received axicabtagene-ciloleucel (axi-cel) with CRS/ICANS prophylaxis, including once-daily oral dexamethasone 10 mg. This regimen resulted in 58% of patients experiencing any grade of ICANS and 13% experiencing grade 3 or higher ICANS (4).

Despite prophylaxis, many patients still succumb to high-grade ICANS. The first-line therapy includes corticosteroids and supportive care. However, concerns are rising regarding the usage of steroids, as their cumulative dose negatively correlates with OS and PFS due to the loss of CAR-T cell function and persistence (2). Moreover, it elevates the risks of severe infections (31). Limited options are available for steroid-refractory ICANS, which include Anakinra, chemotherapy (15, 32), and more treatment modalities like dasatinib (33) are under investigation.

In one study, Anakinra was used in combination with steroids for the management of 6 patients with high-grade 3-4 ICANS after CD19 CAR-T cell therapy. Among the patients, 3 reached grade 0-I ICANS, 1 reached Grade II ICANS, and 2 did not respond. Among the responders, the mean cumulative dose of dexamethasone and the median time to ICANS resolution were 1446 mg and 28.5 days, respectively. Moreover, 2 of the initial responders succumbed to ICANS, disease progression, and hemophagocytic lymphohistiocytosis recurrence (34) (**Table 1**). In another study by Gazeau et al. (35), the utility of Anakinra implementation for the management of steroid-refractory ICANS after CD19 CAR-T cell therapy was explored. In this study, 26 patients with B-cell malignancy were recruited. Half of the patients received low-dose Anakinra (100-200 mg per day) and the other half received high-dose Anakinra (8 mg/kg/day) for managing steroid-refractory ICANS. The median ICANS resolution time was 15.5 days for all the patients. Improvement in ICANS was noted in 73% of patients, with higher response rates seen in the high-dose group compared to the low-dose group (100% versus 46%, respectively). The objective response rate and CR rates were 58% and 42% for the low-dose regimen and 77% and 53% for the high-dose regimen, respectively. The non-relapse mortality rate at day 30 was significantly lower in patients treated with high-dose Anakinra compared to low-dose Anakinra (0% versus 69%) (35) (**Table 1**). The drawback of these studies is the prolonged concurrent use of high dose steroids, which makes it difficult to evaluate the contribution of Anakinra to the clinical efficacy. Furthermore, the negative impact of steroids on CAR-T cell function, which could be observed later during the surveillance period, adds complexity to the evaluation. Some concerns regarding the lack of a clear and dramatic clinical effect on neurotoxicity relief by Anakinra, as well as the lack of corticosteroid tapering for managing ICANS, have been reported (39). Some recent studies indicate that the dose of Anakinra could be safely elevated up to 12 mg/kg/day with enhancement of its efficacy (40).

**Table 1:** The clinical trials for the management of steroid-refractory ICANS.

References	Response rate	Median duration	Median CSD*	Treatment
Strati P. et al. (34)	66%	28.5 days	1446 mg Dex	AN (n=6)
Gazeau N et al. (35)	100% 46%	15.5 days N/R N/R	500 mg Dex 610 mg Dex	AN (n=26) HD AN (n=13) LD AN (n=13)
Zurko JC et al. (36)	100%	6 days	713 mg Dex	IT (HC) (n=7)
Yucebay F et al. (37)	71%	11 days	N/R	IT (MTX+HC+Ara-C) (n=7)
Solh M. et al. (38)	100%	4 days	N/R	IT (MTX, Ara-C)+/- AN (n=12)

*CSD, cumulative steroid dose; AN, Anakinra; N/R, not reported; HC, hydrocortisone; HD, high dose 8mg/kg/day; LD, low dose 100-200 mg/day; Dex, Dexamethazone; MTX, methotrexate.

Due to the possibility that other cytokines, in addition to IL-1, could be implicated in the pathogenesis of ICANS, as well as the potential advantage of local control of CNS inflammation over systemic CAR-T cell depletion, treatment options such as IT administration of immunosuppressive drugs have been proposed. Clinical cases of IT therapy have been reported in the past (37, 41, 42), and recently, small clinical studies have shown favorable outcomes in steroid-refractory ICANS (37) (**Table 1**). Yucebay F. et al. reported a small clinical study investigating the IT administration of methotrexate 15mg, cytarabine 40mg, and hydrocortisone 50mg for the management of steroid-refractory or recurring high-grade ICANS following anti-CD19 CAR-T cell therapy. Overall, 5 out of 7 patients (71%) responded to IT chemotherapy, with total resolution of neurotoxicity occurring in a median of 11 days. The median time to IT chemotherapy from onset of neurotoxicity was 11 days, which could contribute to the late resolution of ICANS (37). A recent retrospective study has highlighted the superior efficacy of early IT hydrocortisone administration for steroid-refractory high-grade ICANS compared to late IT hydrocortisone or systemic corticosteroid therapy (36). In this study, the first group of 7 patients with steroid-refractory ICANS received IT hydrocortisone within the first 5 days after high-grade ICANS development. All patients recovered from ICANS, and the 1-year PFS and OS were 57.1% (**Table 1**). However, three of these patients who received additional treatment with anakinra died of infectious complications. The second group, consisting of 8 patients with high-grade ICANS, received late IT or conventional systemic corticosteroid therapy. For this group of patients, the estimated 1-year PFS and OS were 18.8% for all patients and 0% for patients with steroid-refractory ICANS (36). Solh M.M. et al. reported a clinical study that explored the safety and efficacy of IT administration of methotrexate and/or cytarabine for the treatment of ICANS. In this study, twelve patients received one or two doses of IT chemotherapy. All patients received systemic steroids, and 6 patients (50%) received Anakinra as part of their ICANS management. Eleven patients experienced resolution of their ICANS, with a median time to resolution of 2 days. Additionally, five patients had complete resolution within 24 hours of receiving IT chemotherapy. Among the five patients who did not respond to steroids, all experienced resolution of their ICANS symptoms after IT chemotherapy (38) (**Table 1**).

In our case, given that we infused a very small amount of only 1.29 * 10^6^ CAR-T cells and the patient had high-risk disease features, we chose to manage the grade IV ICANS with the first-line IT chemotherapy (methotrexate, Ara-C, and dexamethasone) to avoid high cumulative doses of systemic steroids. The patient responded very rapidly, with complete resolution of ICANS in less than 32 hours after the onset, and the cumulative dose of steroids was only 96 mg. This more targeted approach of ICANS management helped to prolong the persistence of CAR-T cells, resulting in a durable complete response. Although our patient received a significantly lower cumulative dose of steroids compared to those reported in clinical trials (**Table 1**), it is possible that high-dose systemic steroids could also have contributed to his rapid improvement. However, the rapidity of his response suggests that our early intervention played a role in reversing his ICANS. The potential benefits of IT chemotherapy include direct delivery of immunosuppressive drugs into the CNS, leading to rapid biodistribution in the brain, rapid resolution of neurotoxicity, and dose reduction of systemic steroids. However, it’s important to note that there could be contraindications to the procedure, such as coagulopathy, thrombocytopenia, etc., which may make it difficult to perform an IT chemotherapy in every patient.

## Conclusion

CD4+ CAR-T cells may play a role in both the toxicity and efficacy of CAR-T cell therapy even when infused at very low levels. In cases of high-grade ICANS, early IT therapy with methotrexate, Ara-C, and dexamethasone is a viable option to accelerate the resolution of ICANS and reduce the cumulative dose of systemic corticosteroids. Further clinical trials are needed to evaluate the safety and efficacy of this approach, as it could potentially provide a more targeted and effective management strategy for ICANS associated with CAR-T cell therapy.

## Data availability statement

The original contributions presented in the study are included in the article/supplementary material. Further inquiries can be directed to the corresponding author.

## Ethics statement

The studies involving humans were approved by Independent ethical committee of the health care institution “Vitebsk Regional Clinical Oncology Dispensary”. The studies were conducted in accordance with the local legislation and institutional requirements. The participants provided their written informed consent to participate in this study. Written informed consent was obtained from the individual(s) for the publication of any potentially identifiable images or data included in this article.

## Author contributions

MK: Conceptualization, Writing – original draft, Writing – review & editing, Data curation, Supervision, Validation. TS: Writing – original draft, Writing – review & editing. AM: Writing – original draft, Writing – review & editing. DL: Writing – original draft, Writing – review & editing. YS: Writing – original draft, Writing – review & editing. YKh: Writing – original draft, Writing – review & editing. YKo: Writing – original draft, Writing – review & editing. SD: Writing – original draft, Writing – review & editing.
